# CLOCK expression identifies developing circadian oscillator neurons in the brains of *Drosophila *embryos

**DOI:** 10.1186/1471-2202-9-119

**Published:** 2008-12-18

**Authors:** Jerry H Houl, Fanny Ng, Pete Taylor, Paul E Hardin

**Affiliations:** 1 Center for Research on Biological Clocks, Department of Biology, Texas A&M University, 3258 TAMU, College Station, TX 77843, USA; 2 Department of Biology and Biochemistry, University of Houston, 4800 Calhoun, Houston, TX 77204, USA; 3 Department of Neuroscience, Tufts University School of Medicine, 136 Harrison Ave., Boston, MA 02111, USA; 4 Department of Pediatrics, MD Anderson Cancer Center, 1515 Holcombe, Houston, TX 77030, USA

## Abstract

**Background:**

The *Drosophila *circadian oscillator is composed of transcriptional feedback loops in which CLOCK-CYCLE (CLK-CYC) heterodimers activate their feedback regulators *period *(*per*) and *timeless *(*tim*) via E-box mediated transcription. These feedback loop oscillators are present in distinct clusters of dorsal and lateral neurons in the adult brain, but how this pattern of expression is established during development is not known. Since CLK is required to initiate feedback loop function, defining the pattern of CLK expression in embryos and larvae will shed light on oscillator neuron development.

**Results:**

A novel CLK antiserum is used to show that CLK expression in the larval CNS and adult brain is limited to circadian oscillator cells. CLK is initially expressed in presumptive small ventral lateral neurons (s-LN_v_s), dorsal neurons 2 s (DN_2_s), and dorsal neuron 1 s (DN_1_s) at embryonic stage (ES) 16, and this CLK expression pattern persists through larval development. PER then accumulates in all CLK-expressing cells except presumptive DN_2_s during late ES 16 and ES 17, consistent with the delayed accumulation of PER in adult oscillator neurons and antiphase cycling of PER in larval DN_2_s. PER is also expressed in non-CLK-expressing cells in the embryonic CNS starting at ES 12. Although PER expression in CLK-negative cells continues in *Clk*^Jrk ^embryos, PER expression in cells that co-express PER and CLK is eliminated.

**Conclusion:**

These data demonstrate that brain oscillator neurons begin development during embryogenesis, that PER expression in non-oscillator cells is CLK-independent, and that oscillator phase is an intrinsic characteristic of brain oscillator neurons. These results define the temporal and spatial coordinates of factors that initiate *Clk *expression, imply that circadian photoreceptors are not activated until the end of embryogenesis, and suggest that PER functions in a different capacity before oscillator cell development is initiated.

## Background

Most organisms exhibit daily rhythms in physiology, metabolism, and behavior that persist in the absence of environmental cues. In animals, these ~24 hr rhythms are controlled by circadian oscillators that reside in the central nervous system (CNS) and/or peripheral tissues. These oscillators are comprised of interlocked transcriptional feedback loops that regulate rhythmic gene expression within and downstream of the circadian timekeeping mechanism.

In *Drosophila*, the *per*/*tim *and *Clk *feedback loops control rhythmic transcription that peaks around dusk and dawn, respectively (reviewed in [1-3]). The *per*/*tim *feedback loop is initiated during mid-day, when CLK/CYC heterodimers bind E-box sequences to activate *per *and *tim *transcription [4,5]. Although *per *and *tim *mRNAs peak around dusk, phosphorylation of PER and TIM delays their peak accumulation to the late evening and promotes their nuclear localization [6-10]. After entering the nucleus, PER or PER-TIM heterodimers bind CLK to inhibit CLK-CYC-dependent transcription [11-13]. In addition, *clockwork orange *(*cwo*) is also thought to inhibit *per *and *tim *transcription by competing for E-box binding with CLK-CYC [14-17]. PER and TIM are then degraded after dawn, thus relieving transcriptional inhibition. CLK-CYC initiates the *Clk *feedback loop by binding E-boxes to activate *vri *transcription [18]. VRI accumulates in parallel with *vri *mRNA during early evening and binds to V/P-boxes to repress *Clk *transcription [19,20]. Mutants that disrupt CLK-CYC transcriptional activity (*e.g. Clk*^Jrk^, *cyc*^01^) exhibit constitutive high levels of *Clk *mRNA [21], indicating that *Clk *is activated independent of circadian oscillator function. Since CLK-CYC is required to initiate circadian feedback loop function, we hypothesize that the activation of *Clk *and *cyc *during development determines oscillator cell identity.

Locomotor activity rhythms in adults can be synchronized by light-dark cycles in L1 larvae, but not in embryos, which indicates that the circadian oscillator is only functional after hatching [22]. Circadian oscillator cells are present in LN_v_s, DN_1_s and DN_2_s from L1 larval brains based on rhythmic expression of PER and TIM [23]. Since entrainment of oscillators to light is TIM dependent, and TIM accumulates in concert with PER about 6–8 h after their respective mRNAs (reviewed in [1-3]), *per *and *tim *transcription are expected to be initiated during embryogenesis. Indeed, *per *mRNA is detected in the central nervous system (CNS) of embryos [24,25], which implies that CLK and CYC accumulate in presumptive oscillator cells during embryonic development. To understand oscillator cell development in *Drosophila*, the spatial and temporal expression of CLK and PER was determined during embryogenesis.

In our previous studies, CLK GP47 antibody revealed CLK expression in circadian oscillator and non-oscillator cell nuclei from adult heads at all times of day [26]. Using a newly generated CLK antibody we show here that CLK is expressed exclusively in circadian oscillator cells, and that detection of CLK in non-oscillator cells in a previous study was due to cross-reactivity with DACHSHUND (DAC). During embryonic development PER is first expressed in the ventral nerve chord (VNC) at ES 12 and then the brain at ES 14, whereas CLK is not detected until ES 16 in brain cells that lack PER expression. These CLK-expressing brain cells correspond to LN_v_s, DN_1_s and DN_2_s, and by the end of ES 16 or early ES 17 PER is detected in LN_v_s and DN_1_s but not DN_2_s. These results demonstrate that presumptive brain oscillator cells are present before functional oscillators are detected around the transition to larval life, suggest that the delayed appearance of PER accumulation in presumptive embryonic DN_2_s gives rise to the antiphase cycling of PER in larval DN_2_s compared to LN_v_s and DN_1_s, and imply that PER has a clock-independent function in the VNC and brain in non-oscillator cells of embryos.

## Results

### CLK expression is detected only in oscillator neurons

**Figure 1:**
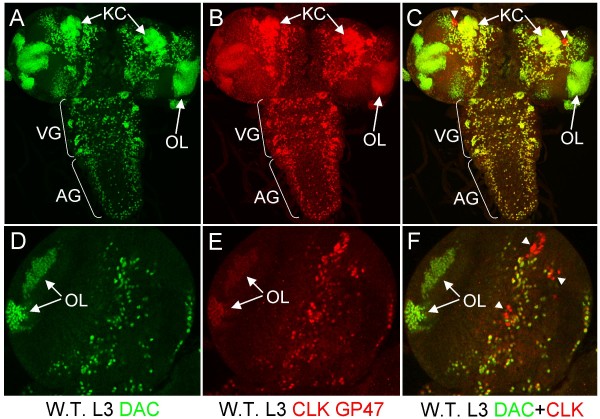
**DAC is co-expressed with CLK in the CNS of L3 larvae**. L3 larval brains were dissected and fixed at ZT1, immunostained with CLK and DAC antibodies, and imaged by confocal microscopy. (A-C) Projected Z-series images of the CNS, where dorsal is at the top. (A) DAC expression in the CNS. Arrows denote DAC immunoreactivity in Kenyon cells (KC) and the optic lobe (OL). (B) CLK expression in the same brain as panel A. Arrows denote CLK immunoreactivity in KCs and the OL. (C) Superimposed dual laser image of DAC and CLK immunostaining in the same larval CNS as panels A and B. Co-localization of DAC (green) and CLK (red) is seen as yellow. Arrowheads, CLK positive/DAC negative cells. (D-F) Magnified 1 μm optical section through the left hemisphere of an L3 larval brain. OL, optic lobe. (D) DAC immunostaining. (E) CLK immunostaining. (F) Superimposed dual laser image of DAC and CLK immunostaining. Arrowheads, CLK positive/DAC negative cells. Images are representative of three independent experiments. Six or more larval CNSs were examined in each experiment. Z-series images are projections over 32 optical sections at 2.5 μm per optical section.

**Figure 2:**
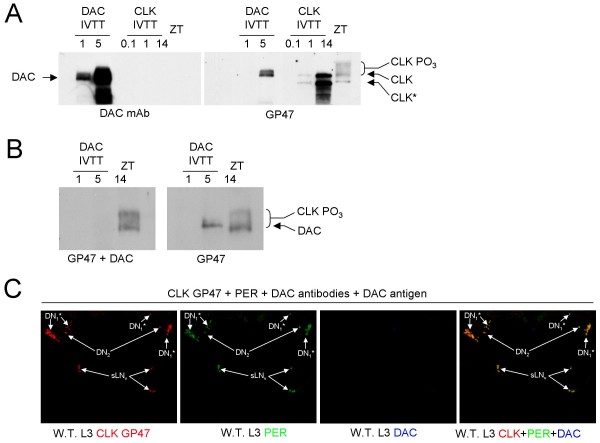
**CLK GP47 antiserum cross-reacts with DAC**. (A) Western blots containing 1.0 μl and 5.0 μl of *in vitro *transcribed and translated DAC (IVTT DAC), 0.1 μl and 1.0 u μl of *in vitro *transcribed and translated CLK (IVTT CLK), and 100 μg of head extract from flies collected at ZT14 probed with either DAC monoclonal antibody (DAC mAb) (left) or CLK GP47 antiserum (GP47) (right). (B) Western blots of samples as denoted in panel A probed with CLK GP47 and 10 μg of purified DAC (GP47 + DAC) (left) or CLK GP47 alone (GP47) (right). (C) Dissected CNSs from wild-type L3 larvae collected at ZT21 were incubated with CLK GP47 antiserum, PER antiserum, DAC monoclonal antibody and 10 μg of purified DAC antigen. A 32 μm projected Z-series image of CLK GP47 IR alone (W.T. CLK), PER IR alone (W.T. PER), DAC IR alone (W.T. DAC), and merged CLK GP47, PER and DAC IR (W.T. merged). Arrows denote brain oscillator cells. s-LN_v_, small ventral lateral neurons; DN_2_, dorsal neuron 2 s; DN_1_*, dorsal neuron 1 s plus additional brain oscillator neurons that may include DN_3_s, l-LN_v_s and LN_d_s.

**Figure 3:**
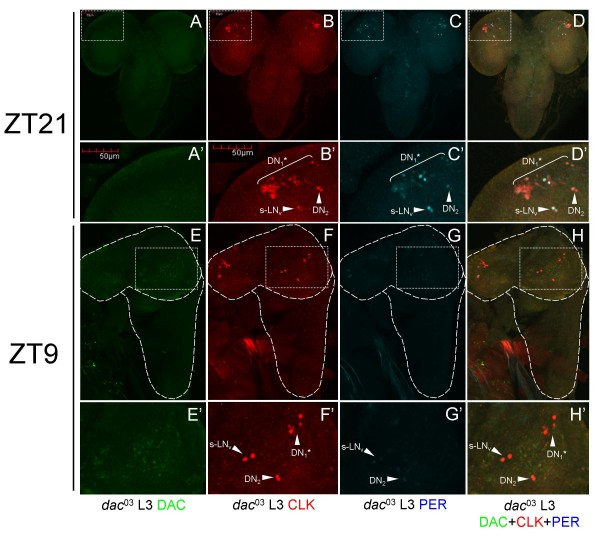
**DAC is required for CLK cross-reactivity in non-oscillator cells**. CNSs were dissected from *dac*^03 ^mutant L3 larvae collected at ZT21 and ZT9, immunostained with DAC, CLK GP47, and PER antibodies, and imaged by confocal microscopy. (A-D, E-H) Projected Z-series images of the CNS from L3 larvae are shown, where dorsal is at the top. (A-D') CNS from an L3 larva collected at ZT21 was imaged for DAC (A), CLK (B), PER (C) or DAC, CLK and PER (D) immunostaining. Boxed region in panels A-D is magnified in panels A'-D', respectively. (B'-C') Brackets denote CLK and/or PER IR in DN_1_* neurons, and arrowheads denote CLK and/or PER IR in s-LN_v _and DN_2 _neurons. DN_1_*, s-LN_v_, and DN_2 _neurons are as defined in the legend for Fig. 2. Co-localization of CLK (red) and PER (blue) appears as white. (E-H') CNS from an L3 larva collected at ZT9 was imaged for DAC (E), CLK (F), PER (G) or DAC, CLK, and PER (H) immunostaining. Boxed region in panels E-H is magnified in panels E'-H', respectively. (F'-H') Arrowheads denote CLK and/or PER IR in DN_1_*, s-LN_v_, and DN_2 _neurons. Images are representative of three independent experiments at each time point. At least three larval CNSs were examined for each experiment. Z-series images are projections over 33 optical sections at 2.5 μm per optical section.

We previously demonstrated CLK immunostaining in all circadian oscillator cells and some non-oscillator cells in adults [26]. One group of non-oscillator cells that showed CLK immunostaining was Kenyon Cells (KCs), which are involved in olfactory learning and memory [27]. To characterize CLK immunostaining during development, we co-stained L3 larval CNSs with CLK, oscillator cell marker PER, and the KC cell marker DAC [23,28-30]. As expected, we observed CLK staining in every PER-expressing cell, but also detected CLK in every DAC-expressing cell (Fig. 1). This surprising correspondence between CLK and DAC expression in non-oscillator cells suggests a relationship between *Clk *and *dac *activation: *Clk *and *dac *are activated by the same activator, *Clk *activates *dac*, or *dac *activates *Clk*. Alternately, CLK and DAC co-immunostaining could also result from cross-reactivity of CLK antiserum to DAC, though little, if any, sequence identity is evident between these two proteins (data not shown). Because CLK and DAC run at a similar apparent molecular weight of ~130 kDa on western blots [13,31], we tested whether our CLK antiserum (GP47) cross-reacted with *in vitro *translated DAC. As expected, GP47 detected CLK on western blots, but this CLK antiserum also detected DAC (Fig. 2A). Competition with purified DAC protein blocked GP47 detection of DAC on western blots (Fig. 2B), confirming DAC cross-reactivity. Likewise, incubating L3 CNSs with purified DAC specifically blocks GP47 immunostaining in DAC expressing non-oscillator cells (Fig. 2C). GP47 antiserum detects more than the canonical 4 to 5 LN_v_s, 2 DN_2_s, and 2 DN_1_s in L3 brains blocked with purified DAC. Although these additional cells could be due to incomplete blocking by DAC, they may also represent DN_3_s, LN_d_s, and/or l-LN_v_s, which have been detected previously in L3 brains [23,32]. CLK immunostaining in non-oscillator cells is also eliminated in the CNS of *dac*^03 ^mutant larvae (Fig. 3), which lack DAC protein [33]. Taken together, these results demonstrate that CLK immunoreactivity (IR) in non-oscillator cells is due to cross-reactivity with DAC, which implies that CLK expression is limited to oscillator cells.

**Figure 4:**
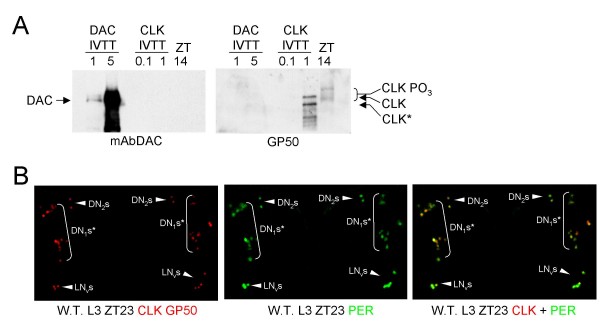
**CLK GP50 antibody does not cross-react with DAC and detects CLK IR only in oscillator cells**. (A) Western blots containing 1.0 μl and 5.0 μl of *in vitro *transcribed and translated DAC (IVTT DAC), 0.1 μl and 1.0 μl of *in vitro *transcribed and translated CLK (IVTT CLK), and 100 μg of head extract from flies collected at ZT14 probed with either DAC monoclonal antibody (mAbDAC) (left) or CLK GP50 antiserum (GP50) (right). (B) Wild-type L3 larval brains were dissected and fixed at ZT23, immunostained with CLK and PER antibodies, and imaged by confocal microscopy. Images show an 18 μm Z-series projection of the CNS, where dorsal is at the top. CLK GP50 (left panel), PER (Middle panel) or merged CLK GP50 + PER (right panel) immunostaining in DN_1_* neurons (brackets) and DN_2 _or s-LN_v _neurons (arrowheads) in both brain hemispheres. DN_1_*, s-LN_v_, and DN_2 _neurons are as defined in the legend for Fig. 2. Co-localization of CLK (red) and PER (green) is shown as yellow. All images are representative of three or more independent experiments.

**Figure 5:**
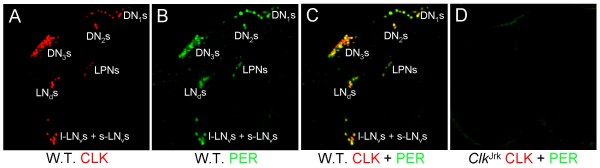
**CLK GP50 antibody only detects oscillator cells in adult brains**. Brains were dissected from adults collected at ZT21, immunostained with CLK GP50 and PER antisera, and imaged by confocal microscopy. (A-C) A 66 μm projected Z-series image of a wild-type adult fly brain, where lateral is left and dorsal is top. CLK (A), PER (B) and CLK + PER (C) IR is detected in dorsal neurons (DN_1_s, DN_2_s, DN_3_s), lateral posterior neurons (LPNs), and lateral neurons (s-LN_v_s + l-LN_v_s). Co-localization of CLK (red) and PER (green) is shown as yellow. (D) A 64 μm projected Z-series image of a *Clk*^Jrk ^adult fly brain, where lateral is left and dorsal is top. No CLK or PER staining is detected. All images are representative of three or more independent experiments.

**Figure 6:**
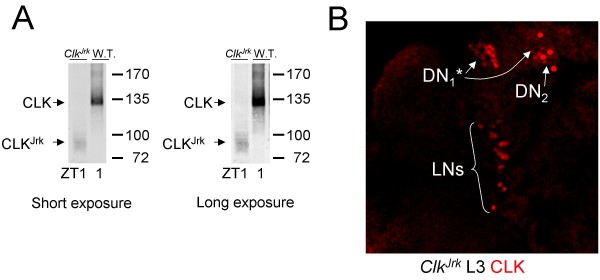
**Truncated CLK^Jrk ^protein accumulates to low levels**. (A) Western blot containing 100 μg of head extract from *Clk*^Jrk ^or wild-type flies collected at ZT1 and probed with CLK GP50 antiserum. Full length CLK and truncated CLK^Jrk ^proteins are denoted in short (left) or long (right) exposures of the blot. (B) Brains from *Clk*^Jrk ^L3 larvae were dissected and fixed at ZT1, immunostained with a lower dilution of CLK GP50 antibody (diluted 1:1000 instead of 1:3000), and imaged by confocal microscopy. Images show a 28 μm Z-series projection of a right brain hemisphere, where dorsal is at the top. CLK GP50 immunostaining is detected in DN_1_*, DN_2 _and LN cells. DN_1_* and DN_2 _cells are as defined in the legend for Fig. 2, and LN cells include s-LN_v_s, l-LN_v_s and LN_d_s. All images are representative of three or more independent experiments.

Additional CLK antisera that we had generated were tested for cross-reactivity to DAC on western blots, and CLK antiserum GP50 showed no cross-reactivity to DAC (Fig. 4A). Based on the lack of CLK IR in non-oscillator cells from *dac*^03 ^mutant larvae and L3 CNSs blocked with DAC antigen, we expected GP50 to detect CLK IR only in oscillator cells. Indeed, CLK and PER co-immunostained several clusters of cells including sLN_v_s, DN_2_s and DN_1_s in L3 larvae (Fig. 4B), but do not detect cells elsewhere in the CNS (compare CLK GP50 immunostaining with GP47 immunostaining in Fig. 1B, E), showing that CLK GP50 antiserum specifically detects oscillator cells in L3 brains. Four additional clusters of brain oscillator neurons are present in adult brains: LN_d_s, l-LN_v_s, LPNs and DN_3_s [34,35]. Brains from adults collected at ZT23 were co-immunostained with GP50 and PER to determine if CLK expression is limited to oscillator neurons. CLK IR is also detected exclusively in oscillator cells from adult brains and is reduced or eliminated in brains from *Clk*^Jrk ^adults (Fig. 5), which express very low levels of truncated CLK protein (Fig. 6). These results demonstrate that GP50 specifically detects CLK, and that CLK is expressed exclusively in circadian oscillator cells in wild-type adult brains. Given that CLK is detected specifically in brain oscillator neurons in adults and L3 larvae, we used CLK GP50 antiserum to determine when brain oscillator neurons first appear during development.

### PER is expressed before CLK during embryogenesis

**Figure 7:**
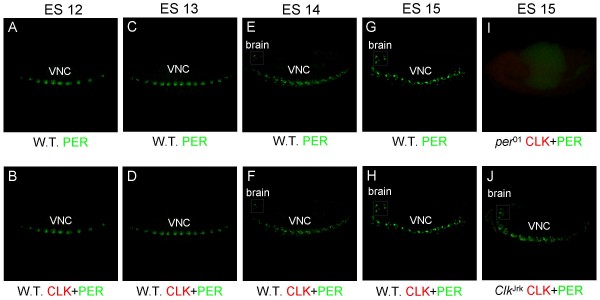
**PER and CLK expression in ES 12 – ES 15 embryos**. 0 h – 24 h wild-type (W.T.), *per*^01^, or *Clk*^Jrk ^embryos were collected at CT33, immunostained with CLK and PER antisera, and imaged by confocal microscopy. (A, B) 12 μm Z-series projection of PER (A) or CLK + PER (B) IR in the VNC of a W.T. embryo during ES 12. (C, D) 20 μm Z-series projection of PER (C) or CLK + PER (D) IR in the VNC of a W.T. embryo during ES 13. (E, F) 18 μm Z-series projection of PER (E) or CLK + PER (F) IR in the brain (box) and VNC of a W.T. embryo during ES 14. (G, H) 30 μm Z-series projection of PER (G) or CLK + PER (H) IR in the brain (box) and VNC of a W.T. embryo during ES 15. (I) 42 μm Z-series projection of PER (E) or CLK + PER (F) IR of a *per*^01 ^embryo during ES 15. (J) 22 μm Z-series projection of PER (E) or CLK + PER (F) IR in the brain (box) and VNC of a *Clk*^Jrk ^embryo during ES 15. Co-localization of CLK (red) and PER (green) is shown as yellow. All images are representative of three or more independent experiments.

To determine whether CLK is expressed in presumptive brain oscillator cells, embryos between 0 h and 24 h old were collected at CT33 and co-immunostained with PER and CLK. PER IR is detected in a segmented pattern along VNC as early as ES 12 (Fig. 7A, B), consistent with previous *in situ *hybridization results in embryos [24,25]. PER in the VNC encompasses more cells, increases in intensity, and expands into the brain by ES 15 (Fig. 7C–H). Loss of PER IR in *per*^01 ^mutant confirms that this IR represents true PER expression (Fig. 7I). Surprisingly, no CLK IR is detected in PER-expressing cells, indicating that *per *is not activated by CLK-CYC during these early developmental stages. The CLK-CYC independent activation of *per *is similar to the situation in ovaries, where PER expression is not associated with circadian oscillator function [36]. To ensure that CLK expression below detectable levels does not activate PER at ES 12–15, *Clk*^Jrk ^embryos were immunostained for PER. These *Clk*^Jrk ^embryos show PER staining in the brain and VNC identical to that in wild-type embryos at ES 15 (Fig. 7J).

### CLK expression preceeds PER expression in presumptive brain oscillator cells during embryogenesis

**Figure 8:**
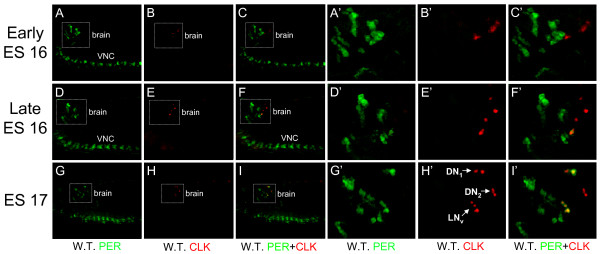
**CLK and PER expression in embryos during ES 16 and ES 17**. 0 h – 24 h wild-type (W.T.) embryos were collected at CT33, immunostained with CLK and PER antisera, and imaged by confocal microscopy. Anterior is to the left and dorsal is on the top. (A-C) 58 μm Z-series projection of PER (A), CLK (B) or CLK + PER (C) IR in the brain (box) and VNC during early ES 16. (A'-C') Magnified view of the boxed brain regions in panels A-C, respectively. (D-F) 18 μm Z-series projection of PER (D), CLK (E) or CLK + PER (F) IR in the brain (box) and VNC during late ES 16. (D'-F') Magnified view of the boxed brain regions in panels D-F, respectively. (G-I) 20 μm Z-series projection of PER (G), CLK (H) or CLK + PER (I) IR in the brain (box) and VNC during ES 17. (G'-I') Magnified view of the boxed brain regions in panels G-I, respectively. Co-localization of CLK (red) and PER (green) is shown as yellow. All images are representative of three or more independent experiments.

Early in ES 16, weak CLK immunoreactivity is first detected in brain cells that do not express PER (Fig. 8A–C). During mid to late ES 16, CLK expression becomes stronger and expands to additional cells in the dorsal brain (Fig. 8D–F). In addition, PER starts to be detected in CLK positive cells in the ventral portion of the brain (Fig. 8F). By ES 17, CLK positive cells form three distinct clusters in each brain hemisphere, with two dorsal cells, four ventral cells, and two cells between and slightly posterior to these dorsal and ventral clusters (Fig. 8G–I). The positions of these CLK positive cells are reminiscent of oscillator cells in larvae; a dorsal cluster of DN_1_s, a ventral cluster of s-LN_v_s, and a medial cluster of DN_2_s [23,37].

Although PER is expressed in some CLK-positive dorsal brain neurons, two cells situated between the most dorsal and ventral CLK-expressing brain cells show little or no PER expression (Fig. 8I). Based on their location, these CLK positive/PER negative cells likely correspond to DN_2_s. In larvae, PER cycling in DN_2_s is antiphase compared to LNs and DN_1_s [23], which is consistent with the absence of PER expression in the presumptive DN_2_s of embryos during the late night and early morning. These results suggest that CLK is expressed in LN_v_s, DN_1_s, and DN_2_s starting at ES 16, followed by PER expression in DN_1_s and LN_v_s during late ES 16 and ES 17. The timeline for CLK and PER expression in embryos is the same whether they are collected at CT49 (1 hour after subjective dawn) or CT37 (1 hour after subjective dusk) (data not shown), indicating that CLK and PER expression are controlled developmentally and are not influenced by the time at which embryos were laid during the circadian cycle.

**Figure 9:**
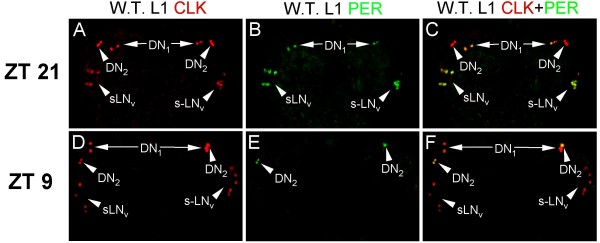
**Antiphase cycling of PER expression in DN_2_s from L1 larvae**. CNSs were dissected from wild-type (W.T.) L1 larvae collected at ZT21 and ZT9, immunostained with CLK and PER antibodies, and imaged by confocal microscopy. Dorsal is at the top. (A-C) A 21 μm projected Z-series image of CLK (A), PER (B), and merged CLK + PER (C) IR in W.T. L1 larvae collected at ZT21. CLK IR is present in DN_1_, DN_2 _and s-LN_v _cells (A, C) and PER IR is detected in DN1 and s-LNv cells (B, C). (D-F) A 22 μm projected Z-series image of CLK (D), PER (E), and merged CLK + PER (F) IR in W.T. L1 larvae collected at ZT9. CLK is present in DN_1_, DN_2 _and s-LN_v _cells (D, F) and PER IR is detected in DN_2 _cells (E, F). Co-localization of CLK (red) and PER (green) is shown in yellow. All images are representative of three or more independent experiments.

To confirm that CLK is expressed in larval LN_v_s, DN_1_s, and DN_2_s, L1 larvae collected at ZT13 and ZT1 were immunostained with CLK GP50 and PER antisera. As expected, CLK is expressed in all PER-expressing cells at ZT21, which includes LN_v_s and DN_1_s (Fig. 9A–C). CLK is also expressed in the presumed DN_2_s, which lack PER expression at this time. In contrast, PER is co-expressed with CLK in DN_2_s at ZT9, but is absent in LN_v_s and DN_1_s (Fig. 9D–F). In addition, little or no PER IR is detected at either time point in the VNC. From these experiments, we conclude that CLK is constantly expressed only in brain oscillator cells from L1 larvae; whereas PER is rhythmically expressed only in oscillator cells from L1 larval brains with high levels at ZT21 in LN_v_s and DN_1_s and at ZT9 in DN_2_s.

## Discussion and conclusion

### CLK is expressed exclusively in oscillator cells

CLK immunostaining was previously detected in all oscillator cells and many non-oscillator cells from adult brains [26]. CLK expression in non-oscillator cells was coincident with that of DAC, which is structurally related to the winged helix/forked-head subfamily of helix-turn-helix DNA binding proteins [38]. Here, we find that CLK IR in non-oscillator cells is due to cross-reactivity between CLK GP47 antiserum and DAC (Fig. 1A–F). We also characterized another CLK antiserum, GP50, and demonstrated that it does not cross-react with DAC on westerns (Fig. 4A). Immunostaining of adult brains with GP50 confirms that CLK is expressed only in oscillator neurons (Fig. 4B). The oscillator cell-specific expression of *Clk *implies that CLK is required for the development and/or function of these cells.

This cell type specificity is consistent with the induction of oscillator cell function, when *Clk *is expressed in ectopic locations [39]. However, CLK expression cannot induce ectopic oscillators in any cell type, suggesting that other factors critical for oscillator function are not activated by CLK or the CLK-dependent developmental programs are incompatible with the development of many cell types. In the loss-of-function *Clk*^Jrk ^mutant, expression of direct CLK-CYC target genes, *per*, *tim*, *vri*, and *Pdp1ε *is abolished [4,18,19], making it difficult to positively identify oscillator cells. Though peripheral oscillator tissues (*e.g. *eye, Malpighian tubule, gut, antenna) apparently develop normally in *Clk*^Jrk ^flies, the loss of oscillator neuron markers in *Clk*^Jrk ^flies makes it difficult to determine whether these neurons are present. One exception to this is l-LN_v_s, which continue to express PDF in *Clk*^Jrk ^flies [40]. Determining whether CLK contributes to oscillator cell development depends on the availability of oscillator cell markers that are expressed independent of CLK-CYC or rescue of a loss-of-function *Clk *mutant, upon CLK induction in adults.

### Oscillator cell development

In adults, CLK-dependent activation of the feedback regulator *per *is required for oscillator function [4,5], thus we expect that *Clk *would be expressed before *per *during development. However, *per *mRNA and protein are expressed in the VNC starting at ES 12 and in the brain at ES 14 (Fig. 7A–F), well before CLK is detected in the brain at ES 16. This early PER expression in the VNC and brain is independent of *Clk *because it persists in the *Clk*^Jrk ^mutant and does not overlap with CLK later in development. The role of PER in CLK negative cells during embryogenesis is unknown, but it is possible that PER modulates transcription by targeting other bHLH-PAS transcription factors (e.g. SINGLE-MINDED) expressed in these cells [41]. Regardless of the role PER plays, the lack of obvious developmental defects in *per*^01 ^flies suggests that *per *is not critical for embryonic development. In larvae, the intensity of PER IR in CLK-negative brain cells and the VNC decreases drastically (Figs. 3, 4, 9), consistent with previous results [19].

CLK can be detected in 2–4 cells in each brain hemisphere starting at early to mid ES 16, and expands during ES 17 to approximately eight CLK-positive cells in each brain hemisphere (Fig. 8). These cells are spatially segregated into three groups that correspond to larval LN_v_s, DN_2_s, and DN_1_s (Fig. 8D–I). PER can be detected in some CLK-positive brain cells starting as early as the end of ES 16 (Fig. 8D), or about 16 h post-fertilization. During ES 17, PER IR increases in intensity and encompasses all four LN_v_s and both DN_1_s (Fig. 8G–I). The 3–6 h delay between CLK detection and PER detection in embryonic brain cells is similar to the delay between the accumulation of *per *mRNA and protein in adults [42,43], and suggests that once CLK-CYC initiates *per *transcription in embryos, PER accumulation is delayed by the same DBT-dependent PER degradation mechanism described in adults. The initiation of molecular oscillator function at ES 17 coincides with the existence of light-entrainable oscillators that mediate behavioral rhythms in adults; a 12 h light pulse ending 6 h before larval hatching didn't synchronize behavioral rhythms of adults, but a 12 h light pulse ending at the time of larval hatching did synchronize behavioral rhythms in adults [22]. The initiation of oscillator function by CLK in embryos is also consistent with *Clk*'s unique ability to initiate oscillator function when expressed in certain ectopic cells [39]. When combined with these previous studies, our results support a model whereby CLK expression initiates circadian oscillator function in brain neurons at ES 16, and these brain neurons go on to control rhythms in locomotor activity in adults.

CLK is expressed in all three groups of oscillator neurons during ES 16 and ES 17, but PER is only detected in LN_v_s and DN_1_s during this time. The delayed onset of PER accumulation in DN_2_s is intriguing, considering that the DN_2_s oscillator is antiphase compared to those in larval LN_v_s and DN_1_s [23]. The antiphase cycling of oscillator in DN_2_s can be brought into phase with oscillators in LN_v_s and DN_1_s by expressing CRY in DN_2_s [44], demonstrating that this antiphase cycling is CRY-dependent. Whether CRY also acts to control antiphase cycling in embryonic DN_2_s will be investigated. In any case, our results demonstrate that antiphase cycling of oscillators in DN_2_s is developmentally regulated and light independent.

The activation and maintenance of *Clk *transcription in developing and adult *Drosophila *is not well understood. The basic zipper protein PDP1ε is involved in maintaining *Clk *activation in adults [19], but does not appear to be the primary *Clk *activator [45]. One approach to defining *Clk *activators in embryos is to first determine which cells within the *Drosophila *embryonic brain express CLK base on co-expression of marker genes [46,47]. Once CLK-expressing cells have been identified, transcriptional activators expressed in these cells can be tested singly or in combination for their ability to activate *Clk*. Identifying factors that activate *Clk *in a cell-specific manner will ultimately reveal determinants of oscillator cell fate.

## Methods

### In vitro translation

The full-length *dac *open reading frame was removed from pUAS-*dac *[48] by digestion with EcoR1 and Xba1 and inserted into the EcoR1 and Xba1 sites of pBluescript KS(-). A plasmid containing the full-length *Clk *open reading frame was described previously (Lee'98). *In vitro *transcription/translation (IVTT) of plasmids containing the complete *dac *or *Clk *open reading frames was carried out using (Promega, L5010) as per manufacturer's instructions by combining the following: 25 μl TNT reticulocyte lysate; 2 μl TNT reaction buffer; 1 μl RNA polymerase; 1 μl complete amino acid mix; 1 μg DNA; to 50 μl with water. Samples were incubated at 30°C for 90 min.

### Protein expression and purification

The complete *dac *open reading frame was removed from pBluescript KS(-) by digestion with EcoR1 and Xho1 and inserted into the EcoR1 and Xho1 sites of pET-28(b). The resulting pET-*dac *plasmid was transformed into BL21(DE3) pLysS cells for protein expression. Cell lysates were purified over a His Trap FF Column (GE, 17-5319-01), and eluants containing DAC were collected and concentrated using an Amicon 100 kDa Concentrator (Millipore, UFC9 100 08). DAC concentration was determined to be ~6.3 μg/μl by spectrophotometric analysis.

### Western blotting

Flies having a normally functioning circadian clock (*white*^1118^) and *Clk*^Jrk ^flies were entrained for at least 3 days in 12 h light: 12 h dark cycles and collected at different Zeitgeber Times (ZTs), where ZT0 is lights-on and ZT12 is lights-off. Fly protein samples were prepared from heads via RBS extraction [13]. Westerns blots were prepared by electrophorescing fly head extract and IVTT DAC and CLK on Criterion pre-cast gels (BioRad) and transferring the gel to Hybond-P membranes (Amersham). Antibodies were used at the following concentrations to probe western blots: GP47, 1:2,000; GP50, 1:5,000; anti-DAC, 1:300. For pre-absorption with DAC, GP47 was incubated with the indicated amount of DAC at 4°C overnight with shaking. Incubation with primary antibodies was done at RT for 1 h for both anti-CLK antibodies and anti-DAC at 4°C overnight. Incubation with secondary antibodies was done at RT for 1 hr at a concentration of 1:1,000 using anti-Guinea pig HRP (Sigma, A7289) or anti-mouse HRP (Sigma, A5278) for the anti-CLK and anti-DAC primary antibodies, respectively. Immnuoblots were visualized with ECL Plus (Amersham).

### Embryo and Larva collection and staging

Wild-type (Canton S) flies were entrained to 12 h light: 12 h dark cycles at 25°C for at least 3 days in egg laying cages containing grape agar plates with yeast paste. Lights were turned off and embryos were collected on fresh plates starting at CT9 and ending at CT33 (9 h after subjective dawn). To determine if the circadian clock affected oscillator cell development or phase, embryos were collected on fresh plates from CT10 to CT37 (1 h after subjective dusk) or from CT22 to CT49 (1 h after subjective dawn). After collection, embryos were fixed and staged based on their morphology [49]. L1 and L3 larvae were collected at different times during LD cycles. Different larval stages were identified based on morphology [50].

### Immunostaining embryos

Wild-type (Canton S) embryos were collected and dechorionated as described [51]. Embryos were fixed with 3.7% formaldehyde in PEM buffer with pH6.9 (0.1 M PIPES pH6.9, 1 mM MgCl_2_, 1 mM EGTA) while shaking, washed with methanol, and then re-hydrated with PBST (1 × PBS, 1% BSA, 0.05% Triton X-100). Primary antibody was diluted in PBST and incubated at 4°C overnight. The primary antibodies and dilutions used were: anti-Guinea pig polyclonal CLOCK GP50 antibody at a 1:200 dilution, and anti-rabbit polyclonal PER (gift from J. Hall) that was pre-absorbed against *per*^01 ^embryos as described [52] at a 1:200 dilution. Following primary antibody incubation, embryos were washed with PBST for 30 minutes at least 6 times at room temperature. Embryos were then incubated with a fluorescently labeled secondary antibody at a 1:200 dilution at 4°C overnight. The following secondary antibodies were used: goat anti-guinea pig Cy3 (Jackson ImmunoResearch) for anti-CLOCK, and goat anti-rabbit Alexa 488 (Molecular Probes) for anti-PER. After secondary antibody incubation, the embryos were washed with PBST for 30 minutes at least 6 times at room temperature. Mounting was done using Vectashield (Vector Labs). Six or more embryos were examined at each stage. Each experiment was repeated at least 3 times with similar results. Each Z-series image is a projection of optical thickness at 2 μm per optical section.

### Immunostaining larval CNSs and adult brains

Dissected adult brains were processed as previously described [26]. CNSs from wild-type L3 larvae were dissected in 1 × PBS with pH7.4. Dissected larval CNSs were fixed with 3.7% formaldehyde, washed, and incubated in the following primary antibodies at 4°C overnight: mouse mAbdac2-3 (1:100 dilution), anti-Guinea pig polyclonal CLK GP50 (1:3,000 dilution), and pre-absorbed anti-rabbit polyclonal PER (1:30,000 dilution). More concentrated CLK GP50 antibody (1:1000 dilution) was used to detect CLK in *Clk*^Jrk ^larve. After primary antibody was removed, the samples were washed, then incubated with fluorescently labeled secondary antibodies (diluted 1:200) at 4°C overnight. The following secondary antibodies were used: goat anti-mouse Alexa 647 (Molecular Probes) for mAbdac2-3, goat anti-guinea pig Cy-3 (Jackson ImmunoResearch Laboratories, Inc.) for anti-CLOCK, and goat anti-rabbit Alexa 488 (Molecular Probes) for PER.

### Confocal microscopy

Embryos, larval CNSs, and adult brains were imaged using a Zeiss LSM310 or an Olympus FV1000 confocal microscope. Serial optical scans were obtained at 2 μm intervals and organized using FV1000 confocal software to generate Z-stack images. Images were processed using Adobe Photoshop.

## Abbreviations

PER: PERIOD protein; CLK: CLOCK protein; CYC: CYCLE protein; DAC: DACHSHUND protein; ES: embryonic stage; ZT: Zeitgeber Time; CT: Circadian Time; IVTT: in vitro transcription/translation; W.T.: wild-type; KCs: Kenyon Cells; CNS: Central Nervous System; VNC: ventral nerve chord; OL: optic lobe; L3: 3^rd ^larval instar; L1: 1^st ^larval instar; LD: 12 h light: 12 h dark; s-LN_v_s: small ventral lateral neurons; l-LN_v_s: large ventral lateral neurons; LN_d_s: dorsal lateral neurons; DN_1_s: dorsal neuron1s; DN_2_s: dorsal neuron 2s; DN_3_s: dorsal neuron 3s.

## Authors' contributions

JHH carried out most of the immunostaining experiments, generated and tested antibodies, participated in the design of the study, and drafted the manuscript. FN defined conditions for the immunostaining experiments, carried out some of the immunostaining experiments, participated in the design of the study and data interpretation, and helped to draft the manuscript. PT carried out the western analysis, generated plasmids for protein overexpression, and purified protein. PEH conceived of the study, and participated in its design and coordination and helped to draft the manuscript. All authors read and approved the final manuscript.

## References

[B1] Hardin PE (2005). The circadian timekeeping system of Drosophila. Curr Biol.

[B2] Hardin PE (2006). Essential and expendable features of the circadian timekeeping mechanism. Curr Opin Neurobiol.

[B3] Yu W, Hardin PE (2006). Circadian oscillators of Drosophila and mammals. J Cell Sci.

[B4] Allada R, White NE, So WV, Hall JC, Rosbash M (1998). A mutant *Drosophila *homolog of mammalian Clock disrupts circadian rhythms and transcription of *period *and *timeless*. Cell.

[B5] Darlington TK, Wager-Smith K, Ceriani MF, Staknis D, Gekakis N, Steeves TD, Weitz CJ, Takahashi JS, Kay SA (1998). Closing the circadian loop: CLOCK-induced transcription of its own inhibitors *per *and *tim*. Science.

[B6] Akten B, Jauch E, Genova GK, Kim EY, Edery I, Raabe T, Jackson FR (2003). A role for CK2 in the *Drosophila *circadian oscillator. Nat Neurosci.

[B7] Kloss B, Price JL, Saez L, Blau J, Rothenfluh A, Wesley CS, Young MW (1998). The *Drosophila *clock gene *double-time *encodes a protein closely related to human casein kinase Iepsilon. Cell.

[B8] Lin JM, Kilman VL, Keegan K, Paddock B, Emery-Le M, Rosbash M, Allada R (2002). A role for casein kinase 2alpha in the *Drosophila *circadian clock. Nature.

[B9] Price JL, Blau J, Rothenfluh A, Abodeely M, Kloss B, Young MW (1998). *double-time *is a novel *Drosophila *clock gene that regulates PERIOD protein accumulation. Cell.

[B10] Martinek S, Inonog S, Manoukian AS, Young MW (2001). A role for the segment polarity gene *shaggy*/GSK-3 in the *Drosophila *circadian clock. Cell.

[B11] Lee C, Bae K, Edery I (1998). The *Drosophila *CLOCK protein undergoes daily rhythms in abundance, phosphorylation, and interactions with the PER-TIM complex. Neuron.

[B12] Lee C, Bae K, Edery I (1999). PER and TIM inhibit the DNA binding activity of a *Drosophila *CLOCK-CYC/dBMAL1 heterodimer without disrupting formation of the heterodimer: a basis for circadian transcription. Mol Cell Biol.

[B13] Yu W, Zheng H, Houl JH, Dauwalder B, Hardin PE (2006). PER-dependent rhythms in CLK phosphorylation and E-box binding regulate circadian transcription. Genes Dev.

[B14] Kadener S, Stoleru D, McDonald M, Nawathean P, Rosbash M (2007). Clockwork Orange is a transcriptional repressor and a new Drosophila circadian pacemaker component. Genes Dev.

[B15] Lim C, Chung BY, Pitman JL, McGill JJ, Pradhan S, Lee J, Keegan KP, Choe J, Allada R (2007). Clockwork orange encodes a transcriptional repressor important for circadian-clock amplitude in Drosophila. Curr Biol.

[B16] Matsumoto A, Ukai-Tadenuma M, Yamada RG, Houl J, Uno KD, Kasukawa T, Dauwalder B, Itoh TQ, Takahashi K, Ueda R, Hardin PE, Tanimura T, Ueda HR (2007). A functional genomics strategy reveals clockwork orange as a transcriptional regulator in the Drosophila circadian clock. Genes Dev.

[B17] Richier B, Michard-Vanhee C, Lamouroux A, Papin C, Rouyer F (2008). The clockwork orange Drosophila protein functions as both an activator and a repressor of clock gene expression. J Biol Rhythms.

[B18] Blau J, Young MW (1999). Cycling *vrille *expression is required for a functional *Drosophila *clock. Cell.

[B19] Cyran SA, Buchsbaum AM, Reddy KL, Lin MC, Glossop NR, Hardin PE, Young MW, Storti RV, Blau J (2003). *vrille*, *Pdp1*, and *dClock *form a second feedback loop in the *Drosophila *circadian clock. Cell.

[B20] Glossop NR, Houl JH, Zheng H, Ng FS, Dudek SM, Hardin PE (2003). VRILLE feeds back to control circadian transcription of *Clock *in the *Drosophila *circadian oscillator. Neuron.

[B21] Glossop NR, Lyons LC, Hardin PE (1999). Interlocked feedback loops within the *Drosophila *circadian oscillator. Science.

[B22] Sehgal A, Price J, Young MW (1992). Ontogeny of a biological clock in *Drosophila melanogaster*. Proc Natl Acad Sci USA.

[B23] Kaneko M, Helfrich-Forster C, Hall JC (1997). Spatial and temporal expression of the *period *and *timeless *genes in the developing nervous system of *Drosophila*: newly identified pacemaker candidates and novel features of clock gene product cycling. J Neurosci.

[B24] James AA, Ewer J, Reddy P, Hall JC, Rosbash M (1986). Embryonic expression of the period clock gene in the central nervous system of Drosophila melanogaster. Embo J.

[B25] Liu X, Lorenz L, Yu QN, Hall JC, Rosbash M (1988). Spatial and temporal expression of the *period *gene in *Drosophila melanogaster*. Genes Dev.

[B26] Houl JH, Yu W, Dudek SM, Hardin PE (2006). Drosophila CLOCK Is Constitutively Expressed in Circadian Oscillator and Non-Oscillator Cells. J Biol Rhythms.

[B27] Davis RL (2005). Olfactory memory formation in Drosophila: from molecular to systems neuroscience. Annu Rev Neurosci.

[B28] Kurusu M, Nagao T, Walldorf U, Flister S, Gehring WJ, Furukubo-Tokunaga K (2000). Genetic control of development of the mushroom bodies, the associative learning centers in the Drosophila brain, by the eyeless, twin of eyeless, and Dachshund genes. Proc Natl Acad Sci USA.

[B29] Martini SR, Roman G, Meuser S, Mardon G, Davis RL (2000). The retinal determination gene, dachshund, is required for mushroom body cell differentiation. Development.

[B30] Noveen A, Daniel A, Hartenstein V (2000). Early development of the Drosophila mushroom body: the roles of eyeless and dachshund. Development.

[B31] Chen R, Amoui M, Zhang Z, Mardon G (1997). Dachshund and eyes absent proteins form a complex and function synergistically to induce ectopic eye development in Drosophila. Cell.

[B32] Kaneko M, Hall JC (2000). Neuroanatomy of cells expressing clock genes in Drosophila: transgenic manipulation of the period and timeless genes to mark the perikarya of circadian pacemaker neurons and their projections. J Comp Neurol.

[B33] Mardon G, Solomon NM, Rubin GM (1994). dachshund encodes a nuclear protein required for normal eye and leg development in Drosophila. Development.

[B34] Helfrich-Forster C, Shafer OT, Wulbeck C, Grieshaber E, Rieger D, Taghert P (2007). Development and morphology of the clock-gene-expressing lateral neurons of Drosophila melanogaster. J Comp Neurol.

[B35] Shafer OT, Helfrich-Forster C, Renn SC, Taghert PH (2006). Reevaluation of Drosophila melanogaster's neuronal circadian pacemakers reveals new neuronal classes. J Comp Neurol.

[B36] Beaver LM, Rush BL, Gvakharia BO, Giebultowicz JM (2003). Noncircadian regulation and function of clock genes period and timeless in oogenesis of Drosophila melanogaster. J Biol Rhythms.

[B37] Helfrich-Forster C (2003). The neuroarchitecture of the circadian clock in the brain of Drosophila melanogaster. Microsc Res Tech.

[B38] Silver SJ, Rebay I (2005). Signaling circuitries in development: insights from the retinal determination gene network. Development.

[B39] Zhao J, Kilman VL, Keegan KP, Peng Y, Emery P, Rosbash M, Allada R (2003). *Drosophila *Clock Can Generate Ectopic Circadian Clocks. Cell.

[B40] Park JH, Helfrich-Forster C, Lee G, Liu L, Rosbash M, Hall JC (2000). Differential regulation of circadian pacemaker output by separate clock genes in *Drosophila*. Proc Natl Acad Sci USA.

[B41] Crews ST, Thomas JB, Goodman CS (1988). The Drosophila single-minded gene encodes a nuclear protein with sequence similarity to the per gene product. Cell.

[B42] Hardin PE, Hall JC, Rosbash M (1990). Feedback of the *Drosophila period *gene product on circadian cycling of its messenger RNA levels. Nature.

[B43] Zerr DM, Hall JC, Rosbash M, Siwicki KK (1990). Circadian fluctuations of *period *protein immunoreactivity in the CNS and the visual system of *Drosophila*. J Neurosci.

[B44] Klarsfeld A, Malpel S, Michard-Vanhee C, Picot M, Chelot E, Rouyer F (2004). Novel features of cryptochrome-mediated photoreception in the brain circadian clock of *Drosophila*. J Neurosci.

[B45] Benito J, Zheng H, Hardin PE (2007). PDP1epsilon functions downstream of the circadian oscillator to mediate behavioral rhythms. J Neurosci.

[B46] Sprecher SG, Reichert H, Hartenstein V (2007). Gene expression patterns in primary neuronal clusters of the Drosophila embryonic brain. Gene Expr Patterns.

[B47] Younossi-Hartenstein A, Nguyen B, Shy D, Hartenstein V (2006). Embryonic origin of the Drosophila brain neuropile. J Comp Neurol.

[B48] Tavsanli BC, Ostrin EJ, Burgess HK, Middlebrooks BW, Pham TA, Mardon G (2004). Structure-function analysis of the Drosophila retinal determination protein Dachshund. Dev Biol.

[B49] Campos-Ortega JA, Hartenstein V (1997). The Embryonic Development of Drosophila melanogaster.

[B50] Roberts DB (1998). Drosophila: A Practical Approach.

[B51] Sullivan W, Ashburner M, Hawley RS (2000). Drosophila Protocols.

[B52] Cheng Y, Hardin PE (1998). Drosophila photoreceptors contain an autonomous circadian oscillator that can function without period mRNA cycling. J Neurosci.

